# High-quality monolayer superconductor NbSe_2_ grown by chemical vapour deposition

**DOI:** 10.1038/s41467-017-00427-5

**Published:** 2017-08-30

**Authors:** Hong Wang, Xiangwei Huang, Junhao Lin, Jian Cui, Yu Chen, Chao Zhu, Fucai Liu, Qingsheng Zeng, Jiadong Zhou, Peng Yu, Xuewen Wang, Haiyong He, Siu Hon Tsang, Weibo Gao, Kazu Suenaga, Fengcai Ma, Changli Yang, Li Lu, Ting Yu, Edwin Hang Tong Teo, Guangtong Liu, Zheng Liu

**Affiliations:** 1 Centre for Programmable Materials, School of Materials Science and Engineering, Nanyang Technological University, Singapore, 639798 Singapore; 2 NOVITAS, Nanoelectronics Centre of Excellence, School of Electrical and Electronic Engineering, Nanyang Technological University, Singapore, 639798 Singapore; 3 Beijing National Laboratory for Condensed Matter Physics, Institute of Physics, Chinese Academy of Sciences, Beijing, 100190 China; 4 National Institute of Advanced Industrial Science and Technology (AIST), Tsukuba, 305-8565 Japan; 5 Department of Physics, Liaoning University, Shenyang, 110036 China; 6 Division of Physics and Applied Physics, School of Physical and Mathematical Sciences, Nanyang Technological University, Singapore, 637371 Singapore; 7 Temasek Laboratories@NTU, Nanyang Technological University, Singapore, 639798 Singapore; 8 Collaborative Innovation Center of Quantum Matter, Beijing, 100871 China; 9 Centre for Disruptive Photonic Technologies, School of Physical and Mathematical Sciences, Nanyang Technological University, Singapore, 637371 Singapore

## Abstract

The discovery of monolayer superconductors bears consequences for both fundamental physics and device applications. Currently, the growth of superconducting monolayers can only occur under ultrahigh vacuum and on specific lattice-matched or dangling bond-free substrates, to minimize environment- and substrate-induced disorders/defects. Such severe growth requirements limit the exploration of novel two-dimensional superconductivity and related nanodevices. Here we demonstrate the experimental realization of superconductivity in a chemical vapour deposition grown monolayer material—NbSe_2_. Atomic-resolution scanning transmission electron microscope imaging reveals the atomic structure of the intrinsic point defects and grain boundaries in monolayer NbSe_2_, and confirms the low defect concentration in our high-quality film, which is the key to two-dimensional superconductivity. By using monolayer chemical vapour deposited graphene as a protective capping layer, thickness-dependent superconducting properties are observed in as-grown NbSe_2_ with a transition temperature increasing from 1.0 K in monolayer to 4.56 K in 10-layer.

## Abstract (web summary)

Two-dimensional superconductors will likely have applications not only in devices, but also in the study of fundamental physics. Here, Wang et al. demonstrate the CVD growth of superconducting NbSe2 on a variety of substrates, making these novel materials increasingly accessible.

## Introduction

Monolayer superconductors provide ideal models for investigating superconductivity in the two-dimensional (2D) limit, as well as superconductor-substrate interplay^1–5^. Strong enhancement of the transition temperature (*T*_c_) has been reported in the monolayer FeSe/SrTiO_3_ system^6, 7^, which indicates that 2D ultra-thin films have the potential to be high-*T*_c_ superconductors. However, the superconductivity in most monolayers (Pb^8, 9^, In^8, 10^, FeSe^6^) only survives on certain substrates, probably due to particular interface bonds^6, 8^. Monolayer NbSe_2_, has recently been recognized as an intrinsic monolayer superconductor, due to the occurrence of superconductivity without the need of a special substrate^4^. NbSe_2_ crystallizes in the same layered hexagonal structure as 2*H*-MoS_2_, where the niobium atoms sit at the center of trigonal selenium prisms. Compared with the bulk, 2D NbSe_2_ exhibits significantly different properties arising from reduced dimensionality, as exemplified by the observation of Ising superconductivity^11^, quantum metallic state^12^, and strong enhancement of charge density wave order^4^.

Defects in an ultrathin superconductor are known to be a critically detrimental factor to intrinsic 2D superconductivity^13, 14^. Monolayer ambient-sensitive^15, 16^ NbSe_2_ is predisposed to receive defects from the substrate and ambient environment. Therefore, the growth of superconducting NbSe_2_ monolayers is a great challenge. Though superconducting NbSe_2_ layers can be mechanically exfoliated from bulk NbSe_2_ crystals, it is not a scalable method. Also, the thickness and size of exfoliated NbSe_2_ flakes cannot be controlled. In the past few years, chemical vapour deposition (CVD) has been widely employed to synthesize ultrathin semiconducting transition metal dichalcogenides (TMDs) such as MoS_2_ and WSe_2_^17–24^. Recently, NbSe_2_ multilayers were prepared by selenizing pre-deposited Nb_2_O_5_ films. However, none of the prepared NbSe_2_ was found to be superconducting^25, 26^, probably due to the large concentration of defects created during growth. Currently, superconducting NbSe_2_ monolayers can only be grown by molecular beam epitaxial (MBE) under ultrahigh vacuum (UHV) and on a dangling bond-free graphene or *h*-BN substrate^3, 27^, in order to minimize environment- and substrate-induced disorders/defects. However, MBE is expensive to perform because it requires a high-priced apparatus and a UHV environment. Furthermore, MBE growth of NbSe_2_ is only available on specific graphene and *h*-BN substrates^3, 27^, and the individual domain size is < 1 µm^3, 27^. Therefore, it is desirable to develop a facile growth method to produce high-quality superconducting NbSe_2_ layers.

In this work, we report the growth of the monolayer superconductor NbSe_2_ at ambient pressure on a variety of substrates. Atomic-resolution annual dark-field scanning transmission electron microscope (ADF-STEM) imaging reveals the atomic structure of the intrinsic defects in the as-grown monolayer NbSe_2_ crystal, such as point defects and grain boundaries, and confirms the low concentration of defects in both mono- and few-layer regions. Transport data indicate that even low concentration of defects exist in CVD-grown NbSe_2_, they will not significantly affect the superconducting properties.

## Results

### Growth of monolayer NbSe_2_ crystals

**Fig. 1:**
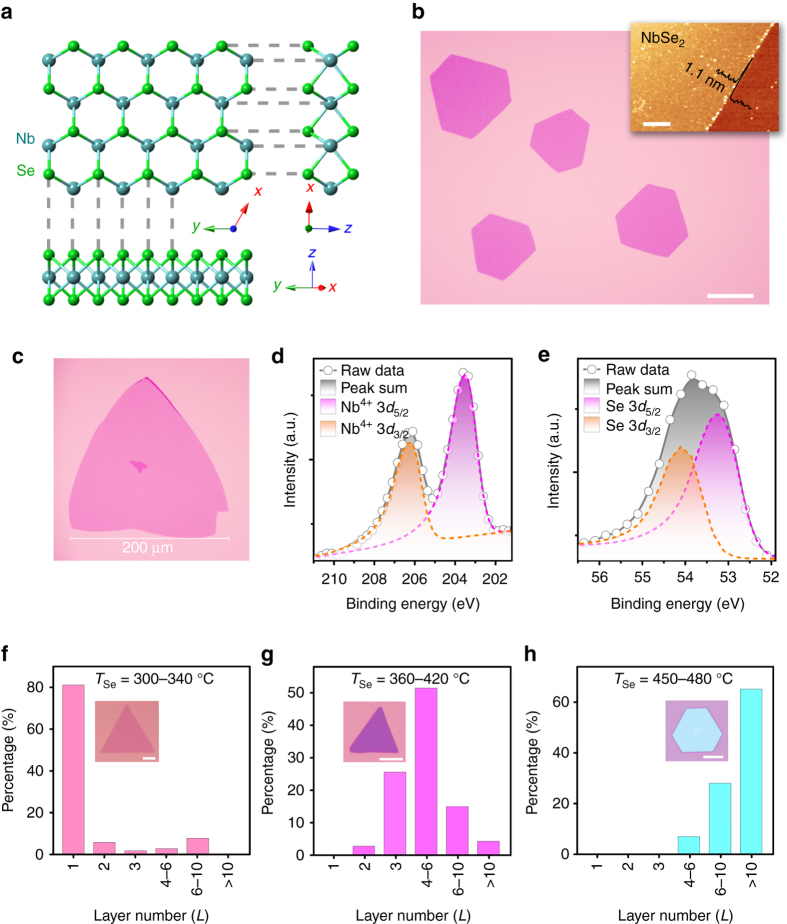
Atomic structure, morphologies, and characterizations of NbSe_2_ crystals. **a** Ball-and-stick model of monolayer 2*H*-NbSe_2_ viewed from three different directions. **b** Optical image of uniform NbSe_2_ crystals deposited on a SiO_2_/Si substrate. *Scale bar*, 40 µm. A representative AFM image (*inset*; *scale bar*, 1 µm) shows the typical thickness is 1.1 nm. **c** A monolayer NbSe_2_ crystal with edge length of 0.2 mm. **d**, **e** X-ray photoemission spectroscopy (*XPS*) spectra of the **d** Nb 3*d* and **e** Se 3*d* peaks from NbSe_2_ crystals deposited on SiO_2_/Si substrate. **f**–**h** Statistic thickness distributions and representative morphologies (*inset*) of NbSe_2_ crystals synthesized with *T*_Se_ setting at **f** 300-340, **g** 360-420 and **h** 450-480 °C, respectively. *Scale bars* from *inset* of **f**–**h** are 20, 5 and 5 µm. Thickness of *inset* crystals of **f**–**h** are 1.1, 5.1 and 16.2 nm

Atomically thin NbSe_2_ crystals were grown on diverse substrates by ambient pressure CVD in a tube furnace (Supplementary Fig. 1). Partially oxidized niobium powder NbO_*x*_ (*x* ≤ 2.5) was chosen as the precursor (Supplementary Fig. 2). Briefly, the powder mixture of NbO_*x*_ and NaCl was loaded in an alumina boat into the center of a fused quartz tube. Diverse substrates (SiO_2_/Si, Si(100), quartz, etc.) were placed 1-3 mm above the powder mixture with the polished side faced down. Selenium powder was located at the entrance of the tube furnace, where the temperature was 300–340 °C during growth. Further details of the experiments are provided in the “Methods” section. Figure 1a shows the crystal structure of monolayer NbSe_2_ viewed from different angles. The as-deposited NbSe_2_ crystals on SiO_2_/Si are typically triangular or hexagonal in shape. Figure 1b shows the optical image of uniform hexagonal NbSe_2_ crystals. A representative atomic force microscopy (AFM) measurement (*inset* of Fig. 1b) indicates the thickness of the NbSe_2_ crystal is around 1.1 nm, which corresponds to a monolayer TMD^20, 28^. As shown in Fig. 1c, the lateral domain size of monolayer CVD-grown NbSe_2_ can reach 0.2 mm, which is ~10^2^ times as large as that of NbSe_2_ prepared by MBE (<1 µm)^3, 27^. In addition to SiO_2_/Si substrates, our CVD method can deposit NbSe_2_ layers on arbitrary selenium-resistant crystalline and amorphous substrates, such as silicon (100), quartz, and CVD graphene (Supplementary Fig. 3). Different substrates may result in NbSe_2_ flakes of varied morphologies (Supplementary Figs. 3 and 4), suggesting that the underlying substrate has an influence on the nucleation and growth of NbSe_2_. Direct growth on diverse substrates makes it feasible to study the NbSe_2_-substrate interaction and related properties.

The chemical states of the as-grown NbSe_2_ samples were examined by X-ray photoemission spectroscopy (XPS) (Supplementary Fig. 5). Figure 1d is the Nb 3*d* core-level spectrum, and the two peaks at 203.4 and 206.1 eV can be assigned to Nb^4+^ 3*d*_5/2_ and Nb^4+^ 3*d*_3/2_ of NbSe_2_^29^. The absence of Nb^5+^ 3*d*_5/2_ and Nb^5+^ 3*d*_3/2_ peaks at higher binding energy (from 207.3 to 211.0 eV) indicates that the sample was not oxidized^30^. The Se 3*d* core levels spectrum can be fitted with Se 3*d*_5/2_ (53.2 eV) and Se 3*d*_3/2_ (54.1 eV) peaks in agreement with the spectra of NbSe_2_ (Fig. 1e)^29, 30^. Furthermore, the absence of Na 1*s* and Cl 2*p* peaks suggests that as-grown NbSe_2_ was not contaminated by the NaCl precursor (Supplementary Fig. 6).

### Tuning the thickness of NbSe_2_ layers

In addition to producing monolayer NbSe_2_, the proposed CVD method is also capable of acquiring NbSe_2_ crystals with different thicknesses from 1.1 to 16 nm, which will be useful for investigating the thickness-dependent properties such as superconductivity, charge–density-wave order and their interplay in the 2D system. The NbSe_2_ layer thickness was found to be sensitive to the heating temperature of selenium source (*T*_Se_). A portable thermocouple thermometer was used to measure the selenium temperature during sample growth. Figure 1f-h shows the thickness distribution statistics and representative morphologies (*inset* of Fig. 1f-h and Supplementary Fig. 7) of NbSe_2_ crystals grown with *T*_Se_ of (f) 300–340, (g) 360–420, and (h) 450–480 °C, respectively, while keeping all other growth parameters identical. Notably, the average thickness of obtained NbSe_2_ increased with *T*_Se_. Monolayer NbSe_2_ crystals were obtained only when selenium was heated to 300–340 °C. In our experimental set-up, relatively higher *T*_Se_ will induce a higher flow rate of the selenium vapour. Therefore, these results indicate that the thickness of NbSe_2_ films highly relies on the flow rate of the selenium vapour. Supplementary Fig. 8 shows AFM images of NbSe_2_ crystals with different thicknesses up to 16.2 nm. Raman spectra of the as-grown crystals show two characteristic peaks of NbSe_2_ (Supplementary Fig. 9), including the in-plane E_2g_ mode at about 250 cm^−1^ and the out-of-plane A_1g_ mode at about 225 cm^−1^. The broad feature at about 180 cm^−1^ is described as a soft mode because of its frequency behavior with temperature^4, 31^. Thickness dependence of the Raman spectra of NbSe_2_ is also shown in Supplementary Fig. 9. For monolayer NbSe_2_, the Raman intensity of both A_1g_ and E_2g_ are very weak at room temperature. With the sample thickness increases, the intensity of A_1g_ and E_2g_ increase significantly.

### Structural characterization

**Fig. 2:**
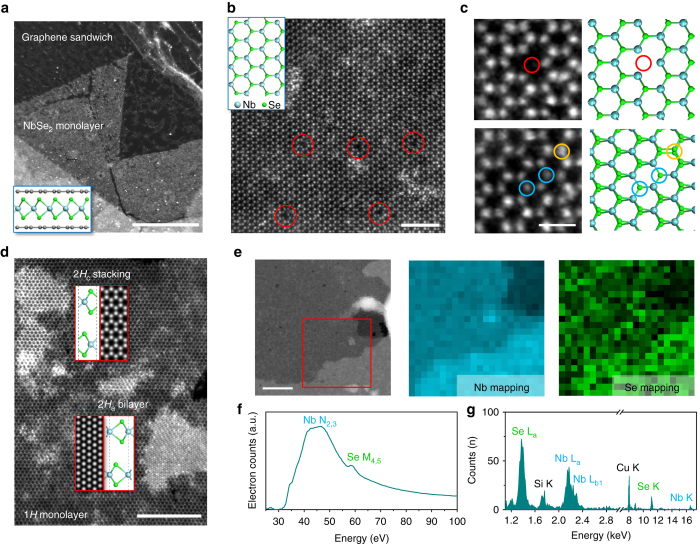
ADF-STEM images, EELS, and EDX characterizations of the as-synthesized NbSe_2_ atomic layers. **a** A low magnified annular dark-field scanning transmission electron microscope (*ADF-STEM*) image showing a large region of monolayer NbSe_2_ encapsulated by the graphene sandwich. The schematic is shown in the *inset*. **b** Atomic resolution ADF-STEM image of the hexagonal NbSe_2_ lattice. Diselenium vacancies are highlighted by *red circles*. The *inset* of panel **b** shows the structural model of 2*H*-NbSe_2_, with *cyan* and *green color* indicating Nb and Se atoms, respectively. **c** Different point defects in monolayer NbSe_2_ and their atomic models. Diselenium vacancy, monoselenium vacancy, and anti-site defect Se_Nb_ are highlighted by *red*, *blue*, and *yellow circles*, respectively. **d** Atomic resolution ADF-STEM image of two bilayer islands in NbSe_2_, showing the coexistence of 2*H*_a_ and 2*H*_c_ stacking sequence. The *insets* are corresponding atomic models and simulated STEM images. **e** STEM image of a large region of NbSe_2_ used for the collection of electron energy-loss spectroscopy (*EELS*) and energy-dispersive X-ray spectrometry (*EDX*) spectra. The collected region is highlighted in *red square*. Nb and Se EELS mapping are provided next to it. **f** Typical EELS and **g** EDX spectra of the region shown in **e**. The Cu signal in the EDX spectra comes from the Cu grid. *Scale bars*, 500 nm (**a**), 2 nm (**b**), 0.5 nm (**c**), 5 nm (**d**), 50 nm (**e**)

In order to examine the quality of the as-grown 2*H*-NbSe_2_ atomic layers, the atomic structure and the chemical composition of the NbSe_2_ layers were characterized by atomic resolution ADF-STEM imaging, electron energy-loss spectroscopy (EELS) and energy-dispersive X-ray spectrometry (EDX). Since a bare NbSe_2_ monolayer film is sensitive to the ambient environment, we transferred the NbSe_2_ flakes grown on SiO_2_ substrate with graphene encapsulation (see “Methods” section), constructing a graphene/NbSe_2_/graphene sandwich structure for STEM imaging. Such structures have been demonstrated to be effective in protecting sensitive monolayer materials from being oxidized^32^. Figure 2a shows a low magnification ADF-STEM image of a large area of monolayer NbSe_2_ sandwiched by graphene (schematic shown in the *inset*), where little oxidization is observed. Figure 2b shows a zoom-in image of the same area with atomic resolution, displaying the hexagonal atomic lattice of alternating bright and dark spots, which corresponds, respectively, to the Se_2_ and Nb atomic columns as indicated in the atomic model, confirming its hexagonal phase. Diselenium vacancy can be directly visualized by their distinguishable contrast within the image, as highlighted by the *red circles*. Since the STEM image contrast is directly related to the atomic number and the number of atoms of the imaged species, more types of intrinsic point defects can be identified by carefully examining the image contrast of each atomic column. Figure 2c shows the atomic resolution image of three major types of point defects found in the monolayer NbSe_2_ film, which are diselenium vacancy (${{\rm{V}}_{{\rm{S}}{{\rm{e}}_{\rm{2}}}}}$, *red*), monoselenium vacancy (V_Se_, *blue*), and anti-site defect where a Se_2_ column replaces the Nb (*yellow*). These intrinsic point defects are similar to those found in MoS_2_ and MoSe_2_ with the same hexagonal phase^33, 34^ grown by CVD method. We can therefore calculate the defect concentration in certain area by counting the number of point defects. The defect concentration in Fig. 2b is estimated to be ~0.18 nm^−2^, which is similar to the case in mechanical exfoliated monolayer materials^35^, demonstrating the sufficiently low defect concentration in the film grown by our CVD method. The defect concentration is similar in multiple layer of NbSe_2_, as shown in Supplementary Fig. 10.

We further study the stacking sequence in the CVD-grown NbSe_2_ sample. Figure 2d shows two stacking orders that coexist in the bilayer region. While the lower bilayer is confirmed to be in 2*H*_a_ stacking, which is a stacking type commonly found in bulk NbSe_2_, where Nb atoms are aligned to each other between the layers (atomic model in the *inset*), the upper bilayer is revealed to be in 2*H*_c_ stacking, similar to the 2*H* stacking in MoS_2_. This is further evidenced by the equal intensity of each atomic column in the hexagonal rings, suggesting all the Nb atoms are aligned with the Se_2_ columns between the layers. Nevertheless, we found the dominating stacking in bilayers is the 2*H*_a_ stacking phase. This result implies that, though 2*H*_c_ stacking is rarely seen in bulk, it can still form in the CVD process presumably due to the weak van der Waal interlayer interaction, which can be overcome by the rapid nucleation process. EDX and EELS were used to identify the chemical constituents of the as-grown layers. Figure 2e shows the region that is used to perform the EDX and EELS experiment. Both EDX and EELS unambiguously show the as-grown NbSe_2_ film only contains Nb and Se without the presence of any other impurities (Fig. 2f, g). Similar defect and stacking structures are also observed in the NbSe_2_ layers that are directly grown on graphene substrate, as shown in Supplementary Fig. 11.

**Fig. 3:**
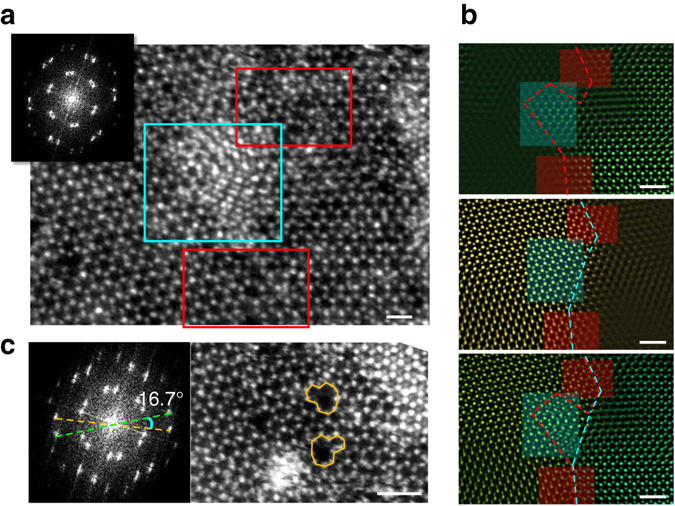
ADF-STEM images of the grain boundary in monolayer NbSe_2_. **a** A tilted grain boundary with misorientation angle of 11°. Both atomically sharp lateral interconnected (*red rectangle*) and vertically stacked (*blue rectangle*) boundary regions are found to coexist. The *inset* shows the fast Fourier transformation (*FFT*) of the image. The distorted polygons are also highlighted in the *red rectangle*. *Scale bar*, 0.5 nm. **b** Selected FFT-filtered image of the two domains and their overlap images. The overlapped image confirms the coexistence of the two types of grain boundaries. *Scale bar*, 1 nm. **c** Similar tilted grain boundary without an overlapping region nearby. The *orange lines* indicate the five-seven dislocation pairs, which is consistent with the theoretical predictions of the grain boundary structure. *Scale bar*, 1 nm

Due to the protection of the graphene sandwich structure, we were able to directly observe the grain boundary structure in the air-sensitive NbSe_2_ monolayer grown on SiO_2_/Si by CVD method. Figure 3a displays a tilted grain boundary with a small misorientation angle (about 11°, determined by fast Fourier transform (FFT) in the *inset*). Interestingly, we found that two types of grain boundary structures coexist, as highlighted by different colored rectangles. The red region indicates the boundary that is atomically sharp, where two domains connects by distorted polygons to accommodate the strain due to the misorientation. The blue region represents another type of boundary, where the two domains overlap with each other, forming a vertically stacked structure. This is further evidenced by the selected FFT-filtered image shown in Fig. 3b, in which the two domains are separated according to the misorientation angles. Although both types of boundaries have been reported previously^36–38^, it is commonly believed that only one of them can be formed depending on the dynamic energy between the growing frontiers of the misoriented domains during the growth process. The coexistence of both types in a single grain boundary structure suggests the formation energy between these two types of structures (atomically sharp and vertically stacked) is similar in NbSe_2_, where their co-growth can be induced by local fluctuation of the growing conditions. Figure 3c shows a similar tilted grain boundary without overlapping regions nearby. The ideal five-seven dislocation pairs are found periodically embedded along the grain boundary (highlighted by *orange outline*), which is similar to the case of other TMD materials^34, 37^ and consistent with the theoretical predictions^39^. In contrast, the polygons are found to be more distorted and did not have a periodic distribution when the overlapping boundary is nearby, as shown in the red rectangular regions in Fig. 3a. This may be due to the alternation of the local strain profile in the atomically sharp grain boundary region induced by the overlapping area, which leads to the random formation of distorted polygons to release the inhomogeneous strain. These results reveal the local atomic structure of the grain boundaries, for the first time, in air-sensitive monolayer NbSe_2_ which is hard to observe due to its easy oxidization without any protection.

### Transport properties of mono- and few-layer NbSe_2_

**Fig. 4:**
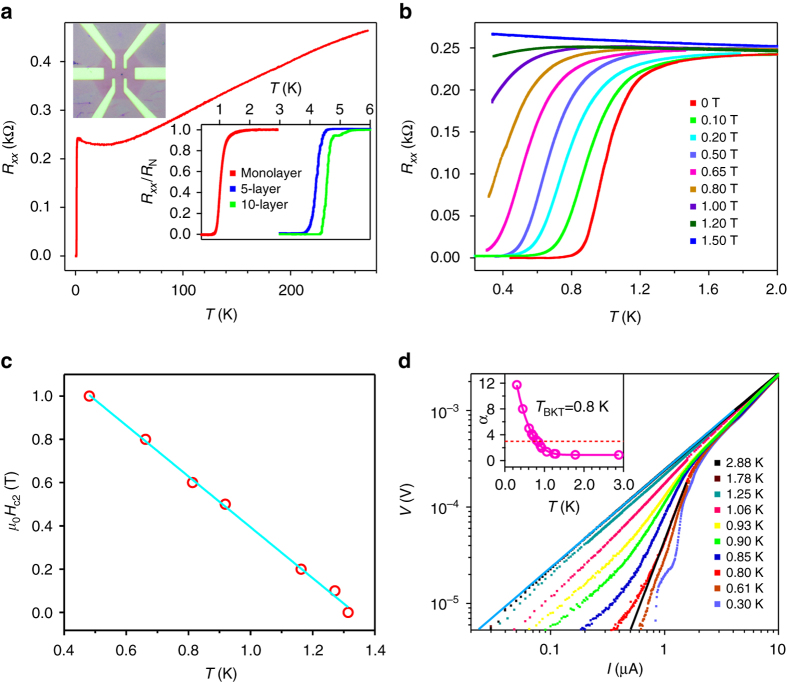
Superconductivity in monolayer NbSe_2_ devices. **a** Temperature dependence of the longitudinal resistance *R*_*xx*_ for sample A—a monolayer NbSe_2_ device. *Upper left inset*: Optical image of a typical graphene protected monolayer NbSe_2_ device. *Lower right inset*: Superconductivity in monolayer, 5-layer and 10-layer NbSe_2_ devices. **b** Superconductivity of sample A in different magnetic fields. **c** Temperature dependence of the upper critical field *H*_c2_. The *solid line* is the linear fit to *H*_c2_. **d** Voltage–current (*V*-*I*) characteristic at different temperatures on a logarithmic scale. The *solid blue line* indicates the Ohmic behavior at high temperature. The *solid black line* represents the expected *V*∝*I* ^3^ behavior at the Berezinskii-Kosterlitz-Thouless (BKT) transition. The *inset* shows the temperature-dependent exponent deduced from the power-law behavior, *V*∝*I* ^*α*^. As indicated by the *red dashed line*, *α* approaches 3 at *T = *0.8 K

To further characterize the quality and dimensionality of as-grown NbSe_2_ crystals, a low-temperature transport experiment was carried out on Hall-bar devices. Because of its air sensitivity^15^, ultrathin NbSe_2_ was first covered with a continuous monolayer graphene film before device fabrication (see “Methods” section). Figure 4a shows the temperature *T* dependence of longitudinal resistance *R*_*xx*_ for sample A—a representative monolayer NbSe_2_ device (see Supplementary Note 1 and Supplementary Fig. 12 for more devices and data) at zero magnetic field. Consistent with previous studies^3, 11, 12^, the sample shows a metallic behavior (d*R*/d*T > *0) at high temperatures. At *T*_onset_ = 1.5 K, *R*_*xx*_ begins to decrease sharply and drops to zero at *T*_zero_ = 0.8 K, indicating the occurrence of superconductivity. Additionally, we find the superconducting transition in our high-quality NbSe_2_ crystals can be tuned by changing the sample thickness. The *lower right inset* of Fig. 4a displays the normalized resistance *R*_*xx*_/*R*_N_ as a function of temperature for NbSe_2_ with different thickness. It is evident that the superconducting transition critical temperature *T*_c_ (0.5*R*_N_) can be tuned from 4.56 K for 10-layer, 4.2 K for 5-layer, to 1.0 K for monolayer. Due to its sensitivity to moisture and oxygen, even when protected by graphene, the superconductivity of NbSe_2_ sample degrades with increasing ambient exposure (Supplementary Fig. 13 and Supplementary Table 1), which is discussed in Supplementary Note 2.

**Fig. 5:**
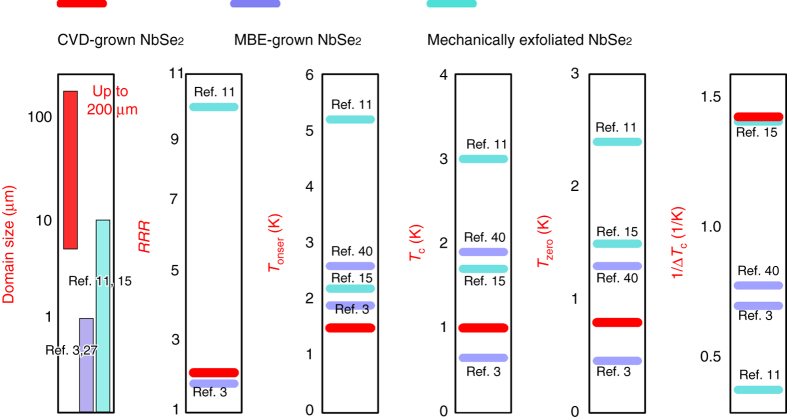
Comparison of the superconducting performance of monolayer NbSe_2_ prepared by CVD and other methods. From *left* to *right*: domain size, residual resistance ratio RRR, *T*_onset_, *T*_c_ (0.5 R_N_), *T*_zero_, and *1/*Δ*T*_c_ for monolayer NbSe_2_ samples prepared with different methods

To compare the quality of the monolayer NbSe_2_ prepared with different methods, the residual resistance ratio (defined as the ratio of the room temperature resistance *R*_300 K_ to the normal-state resistance *R*_N_) RRR, the superconducting transition onset temperature *T*_onset_, the critical temperature *T*_c_ (0.5R_N_), the zero-resistance temperature *T*_zero_, and the inverse of the superconducting transition width *1/*Δ*T*_c_ are summarized in Fig. 5. It is noted that our CVD-grown monolayer NbSe_2_ has a RRR* = *2.0 very close to the reported value for MBE-grown NbSe_2_^3^, but the CVD sample has a *T*_zero_ (0.8 K) almost two times as high as that of MBE NbSe_2_^3^. Though a much higher zero-resistance temperature *T*_zero_ = 1.3 K was reported in Se-capped MBE-grown monolayer NbSe_2_, however, the resistance did not really drop to zero^40^. Besides, the Δ*T*_c_ in the CVD-grown sample is only 0.7 K, which is superior to all reported MBE NbSe_2_^3, 40^ and comparable to the best record in mechanically exfoliated samples^15^. The above data indicate that the CVD-grown NbSe_2_ samples are high quality.

Figure 4b shows the temperature and field dependence of *R*_*xx*_ for sample A (see Supplementary Note 1 for more devices and data) with the field perpendicular to the sample plane. Under a magnetic field, the critical temperature *T*_c_ is defined by where the resistance is 90% of the normal state value *R*_N_. With increasing the strength of magnetic field *B*, *T*_c_ shifts to lower temperatures. Finally, the superconductivity is completely suppressed with a magnetic field of about 1.5 T at *T = *0.3 K. We summarize the *H*_c2_-*T*_c_ phase diagram, where *H*_c2_ is the upper critical field, in Fig. 4c and find a linear relationship between *H*_c2_ and *T*_c_, which is a characteristics of 2D superconductors^11–13^ and can be explained by the standard linearized Ginzburg-Landau (GL) theory^41^, ${H_{{\rm{c}}2}}(T) = \frac{{{\phi _0}}}{{2\pi {\xi _{{\rm{GL}}}}{{(0)}^2}}}\left( {1 - \frac{T}{{{T_{\rm{c}}}}}} \right)$, where ${\xi _{{\rm{GL}}}}$(0) is the zero-temperature GL in-plane coherence length and ${\phi _0}$ is the magnetic flux quantum. As shown in Fig. 4c, a linear fit between *H*_c2_ and *T*_c_ yields ${\xi _{{\rm{GL}}}}$(0) = 18 nm (see Supplementary Note 3 and Supplementary Fig. 14 for more transport data of NbSe_2_ grown on graphene substrate), which is about twice of the bulk value^16^. Based on the normal state resistance *R*_N_ and carrier density *n*_s_ as determined by Hall measurement (Supplementary Note 4 and Supplementary Fig. 15), the mean free path *l*_m_ = 1.3 nm can be obtained, smaller than that in exfoliated sample^12^. On the other hand, the normal state sheet resistance *R*_s_ = 727 Ω (taken at *T*_onset_ = 1.5 K) is much smaller than the quantum resistance *R*_q_ = *h*/4*e* ^2^ ≈ 6.45 kΩ, where *e* is the electron charge and *h* is Planck’s constant, above which a disorder-induced superconductor-insulator transition emerges^42^. The above discussions indicate that our sample is in the low-disorder regime^11, 13, 40^.

For a 2D superconductor with the thickness *d <* ${\xi _{{\rm{GL}}}}$(0), the superconducting phase transition is expected to be of the Berezinskii-Kosterlitz-Thouless (BKT) type^43, 44^. Figure 4d shows voltage–current (*V*-*I*) behavior as a function of temperature on a log-log scale. The *V*-*I* relations change from a linear to a power-law dependence, *V*∝*I* ^*α*^ at *T*_c_ (0.5*R*_N_), which is consistent with expectations for a 2D superconductor based on the theoretical model of the BKT transition. In the *inset* of Fig. 4d, we plot the exponent *α* vs. *T*, which is extracted from the slope of *V*-*I* traces. The BKT transition temperature *T*_BKT_ is estimated to be 0.8 K from where *α = *3 interpolates. The above data confirm that monolayer NbSe_2_ exhibits the characteristics of a true 2D superconductor.

## Discussion

The developed CVD growth technology has enabled the realization of superconductivity in non-UHV grown monolayer materials. Our results also provide a comprehensive understanding of the defect structure, superconducting performance as well as the influence of ambient-induced defects on superconductivity of NbSe_2_. It is evident that even though low concentrations of selenium vacancy-related defects remained in the CVD-grown NbSe_2_, they will not significantly affect the superconducting properties. Our facile CVD technology not only provides an excellent platform for the investigation of many fascinating properties of NbSe_2_, but also holds promise for large-scale synthesis of 2D superconducting NbSe_2_ films for potential device applications. Future developments may also include investigation and understanding of NbSe_2_-substrate interplay and related novel properties, inspired by the research on 2D high-*T*_c_ Fe-chalcogenide superconductors^6, 7, 45^ (more discussion in Supplementary Note 5). More recently, monolayer NbSe_2_ was suggested as a candidate for realizing topological superconducting phase and engineering Majorana fermions^46, 47^. Therefore, the present work offers possibilities for the study of topological physics.

More importantly, our work indicates that a UHV environment is not necessarily required for the growth of monolayer superconductors. In our salt-assisted CVD synthesis of NbSe_2_, the role of salt is particularly important as no nucleation of NbSe_2_ can be observed without salt. It is noted that all niobium oxides have melting points above 1510 °C, which make them difficult to vaporize and react with selenium in the CVD process. The products of reactions between molten salts and metal (Mo^48^, W^28^, and Nb^49^) oxides have been investigated by several groups and found to be metal oxychlorides, which have much lower melting points compared with corresponding metal oxides^28, 48, 49^. Thus, it is suggested that in our CVD process, NaCl reacts with niobium oxides to give volatile niobium oxychloride^28^, therefore increasing the vapour pressure of precursor and facilitating the growth of NbSe_2_. With the proposed salt-assisted CVD method, a number of other 2D and even monolayer superconductors (2*H*-TaS_2_, 2*H*-TaSe_2_, 1*T*-TiSe_2_, and 1*T*-Cu_*x*_TiSe_2_, etc.) could be synthesized by substituting niobium oxides with corresponding metal oxides precursors. Considering that most of the mentioned monolayers have never been synthesized (even by MBE) and investigated, the new growth technology will enrich the research field of 2D TMDs superconductivity greatly.

## Methods

### Atomically thin NbSe_2_ crystals synthesis

We used partially oxidized niobium powder NbO_*x*_ (*x* ≤ 2.5) as precursor for growing NbSe_2_. In the first step, 5 g niobium powder (99.8%, 325 mesh, Alfa Aesar) was loaded into a both-ends-opened quartz tube equipped in a tube furnace. The niobium powder was ignited when furnace was heating to about 680 °C. After 1 min combustion, the niobium powder was rapid cooled to room temperature by moving it to cold zone. X-ray diffraction (XRD) analysis (Supplementary Fig. 2) indicated the obtained power consisted mainly of Nb, Nb_2_O_5_, and NbO.

Ambient pressure CVD growth of NbSe_2_ was conducted in a 2-inch outer diameter fused quartz tube heated by a Lindberg/Blue M (HTF55322C) split-hinge furnace. Supplementary Fig. 1 shows the set-up of the CVD reaction chamber. The partially oxidized Nb powders NbO_*x*_ (0.7 g) together with NaCl powders (0.1 g) were placed in an alumina boat located in the center of the furnace. 285 nm SiO_2_/Si or other substrates were placed 1-3 mm above the powder mixture with the polished side faced down. Selenium powder (2 g) (99.5%, Sigma-Aldrich) was placed at the upstream of the quartz tube, where the temperature ranges from 300 to 460 °C during the reaction. 120 sccm (cubic centimeters per minute) Ar and 24 sccm H_2_ are used as carrier gases. The furnace is heated to 795 °C in 16 min and maintained at that temperature for 13 min to allow the synthesis of NbSe_2_ layers. The furnace was naturally cooled to 680 °C without changing the carrier gases. Then the top cover of the furnace was opened to allow fast cooling of the sample, with carrier gases switched to 250 sccm Ar and 4 sccm H_2_.

### Device fabrication and transport measurements

For fabricating ultrathin NbSe_2_ devices, large-area monolayer CVD graphene was used to cover and protect the thin NbSe_2_ deposited on SiO_2_/Si. First, a rectangular frame consisted by adhesive tape was attached to the poly (methyl methacrylate) (PMMA)-coated graphene grown on Cu foil^50^. Then PMMA/graphene was detached from the Cu substrate by a bubbling transfer method. The PMMA/graphene was rinsed in DI water for several times and then dried in air. Second, the PMMA/graphene film was directly attached to the NbSe_2_ on SiO_2_/Si substrate in an Ar-filled glove box, with the help of a drop of isopropanol added between. PMMA was then removed in acetone to form the graphene/NbSe_2_ stacks. Finally, a fresh film of PMMA was deposited on the prepared graphene/NbSe_2_ layers, by spin-coating at 3 K rpm for 1 min. The PMMA film was cured at 140 °C for 6 min in an Ar glove box. Hall bar device was made by electron-beam lithography and the contact metal (5 nm Cr/50 nm Au) was fabricated by electron-beam deposition.

The transport experiment is carried out in a top-loading helium-3 cryostat in a superconducting magnet. An AC probe current *I*_ac_ = 10 nA at 30.9 Hz is applied from the source to the drain. Then a lock-in amplifier monitors the longitudinal *R*_*xx*_ through two additional electrical contacts.

### Fabrication of graphene/NbSe2/graphene sandwich cell for STEM imaging

First, mono- and few-layer NbSe_2_ crystals were grown on 285 nm SiO_2_/Si substrates. Second, with the same method described earlier in the device fabrication part, a continuous monolayer CVD graphene film was placed on top of the chip to form a graphene/NbSe_2_-stacked heterostructure. Third, a thin PMMA film was spin-coated and cured on the chip, and then the PMMA/graphene/NbSe_2_ film was detached from the SiO_2_/Si substrate in HF. After rinsed in DI water, it was scooped up by a SiO_2_/Si chip predeposited with monolayer graphene film to form a PMMA/graphene/NbSe_2_/graphene structure on SiO_2_/Si substrate. Fourth, the PMMA/graphene/NbSe_2_/graphene film was detached from the substrate by etching SiO_2_ layer in HF. After rinsed in DI water, it was scooped up by a TEM grid. Finally, the graphene/NbSe_2_/graphene sandwich cell was created by removing PMMA in acetone.

### Sample characterizations

XPS spectra were collected on a PHI Quantera II spectrometer using monochromatic Al-Kα (*hυ* = 1486.6 eV) radiation, and the binding energies were calibrated with C 1s binding energy of 284.8 eV. AFM images were taken using the Asylum Research Cypher AFM in tapping mode. Raman spectra were recorded in vacuum by a Witec system with ×50 objective lens and a 2400 lines per mm grating under 532 nm laser excitation. The laser power was fixed at 1 mW. STEM experiments were performed by a low acceleration voltage JEOL 2100F equipped with Delta correctors and GIF quantum spectrometer.

### Data availability

The data that support the findings of this study are available from the corresponding author on reasonable request.

## Electronic supplementary material

Supplementary Information
