# Supplementary material for: High-quality monolayer superconductor NbSe2 grown by chemical vapour deposition
Source: Nat Commun. 2017 Aug 30;8:394. doi: 10.1038/s41467-017-00427-5 (PMC5577275; doi:10.1038/s41467-017-00427-5)
Supplement: Supplementary file 1 — Supplementary Information [file 41467_2017_427_MOESM1_ESM.pdf]

File name: Supplementary Information
Description: Supplementary Figures, Supplementary Notes, Supplementary Tables, Supplementary References

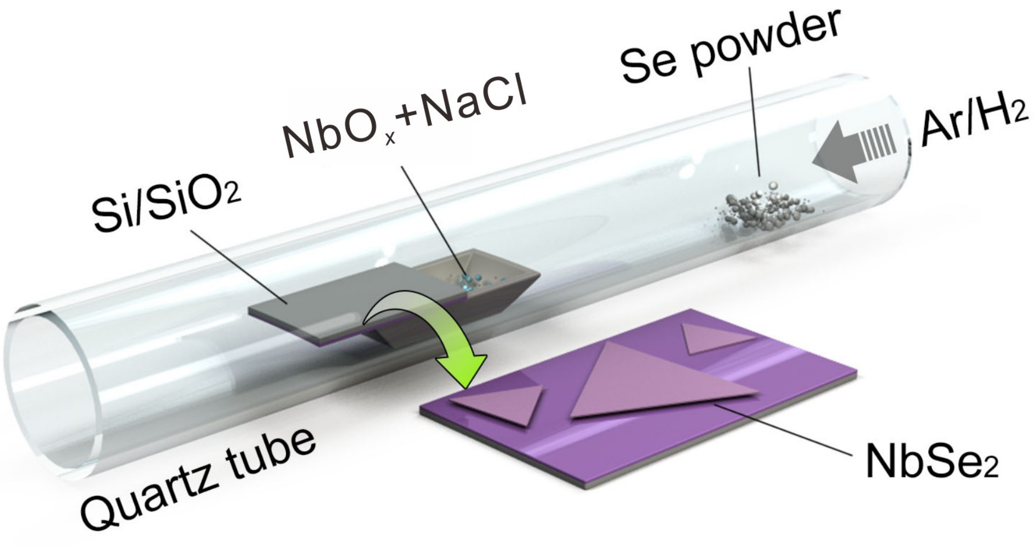

Supplementary Figure 1 Illustration of the reaction chamber for $NbSe_2$ synthesis

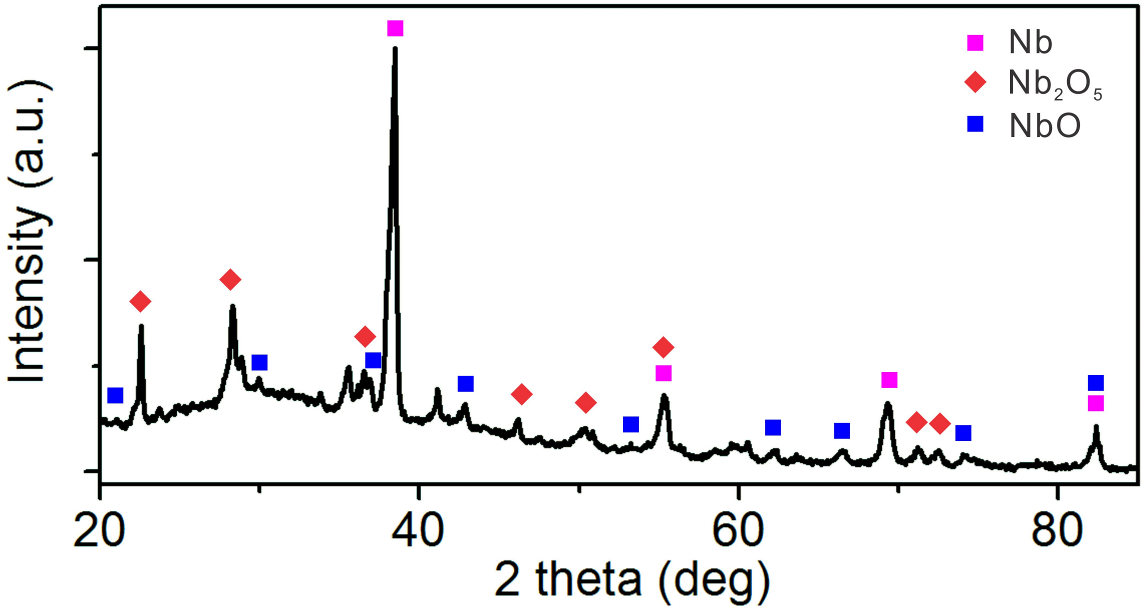

Supplementary Figure 2 XRD pattern of the partially oxidized niobium powder NbO_x ($x \leq 2.5$). After oxidation, the powder contains Nb, Nb_2O_5 and NbO.

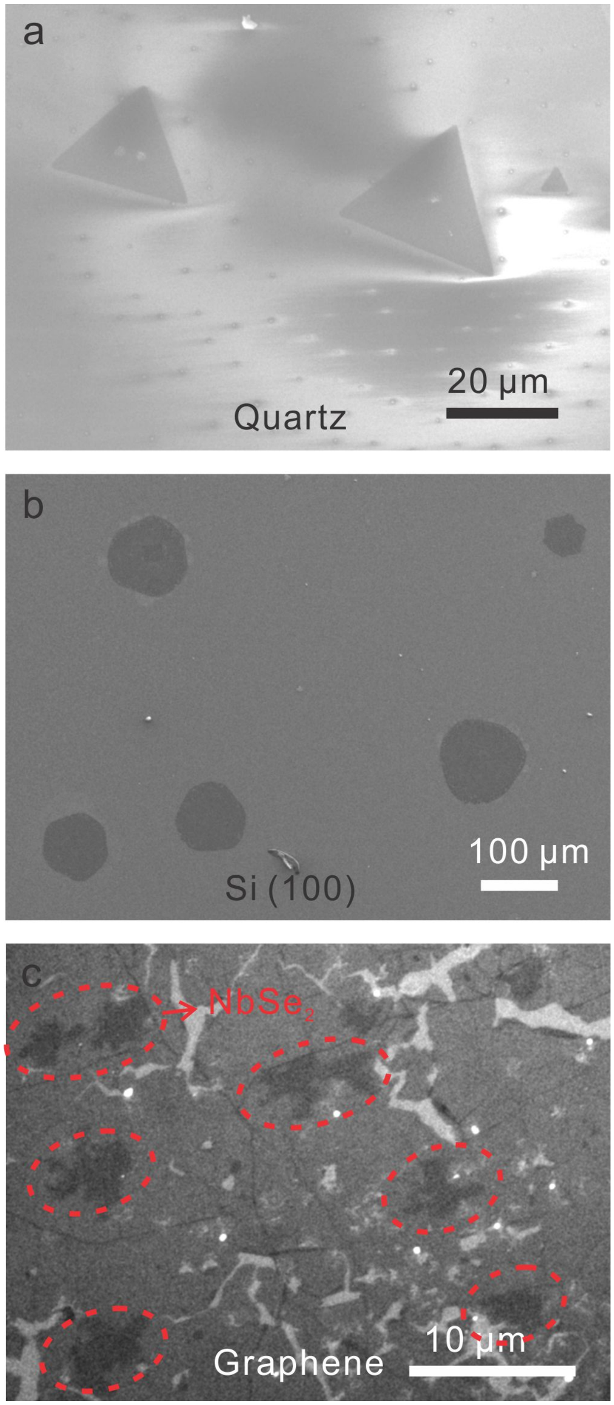

Supplementary Figure 3. SEM morphology of ultrathin $NbSe_2$ layers grown on diverse substrates (a) quartz, (b) Si(100) (c) CVD graphene transferred to SiO_2/Si. This result indicates that $NbSe_2$ layers can be grown on various amorphous or crystalline substrates, different substrates may result in $NbSe_2$ grains with different morphology. The ability to grow on diverse substrates makes it feasible to study the $NbSe_2$-substrate interaction and related properties.

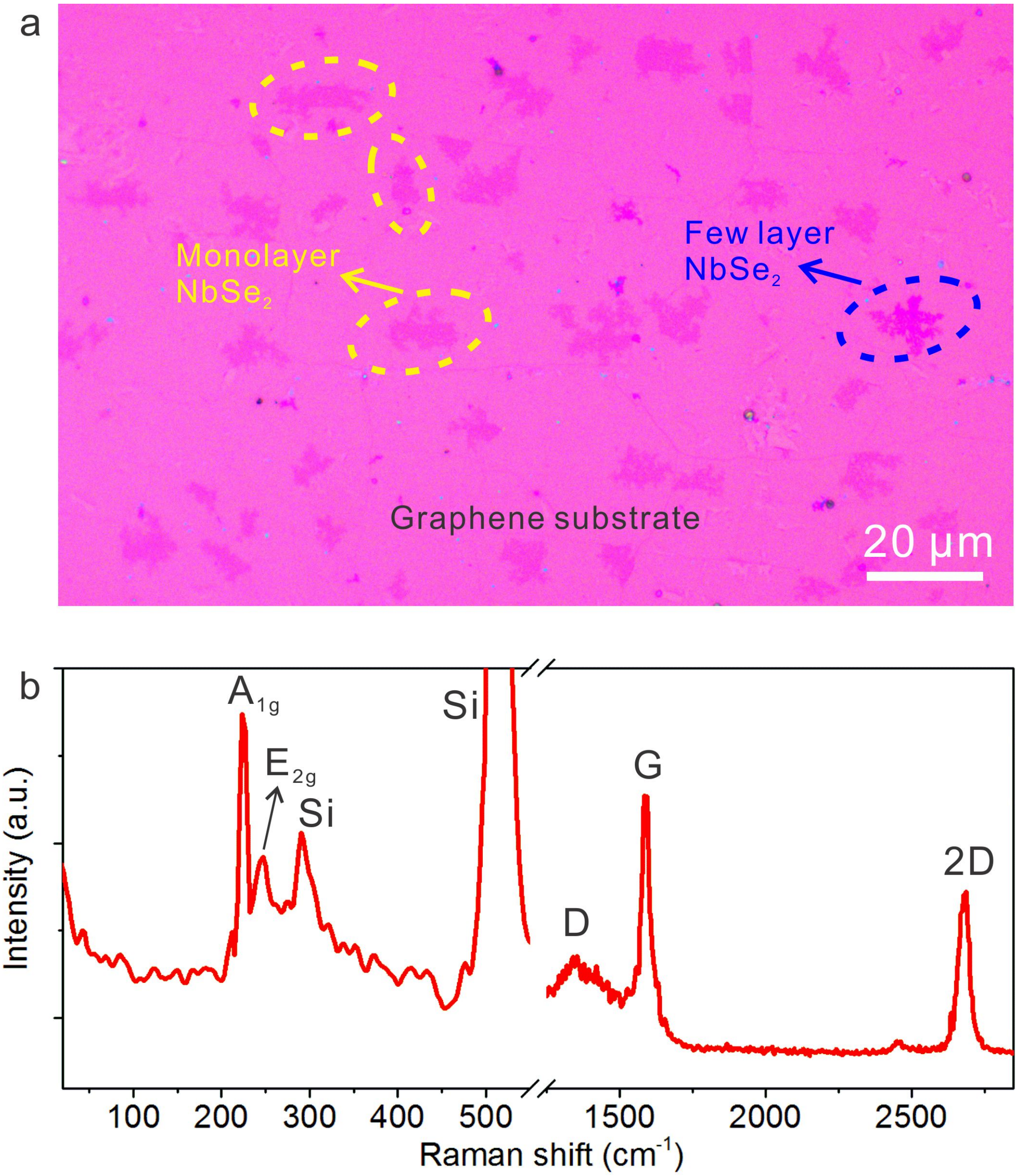

Supplementary Figure 4. Morphology and Raman spectrum of $NbSe_2$ grown on graphene substrates. (a) Optical image of monolayer and few-layer $NbSe_2$ grown on monolayer CVD graphene/Si substrate. $NbSe_2$ grows into irregular shapes on graphene. (b) Raman spectrum of monolayer $NbSe_2$ on monolayer graphene, showing A_{1g} and E_{2g} modes of $NbSe_2$.

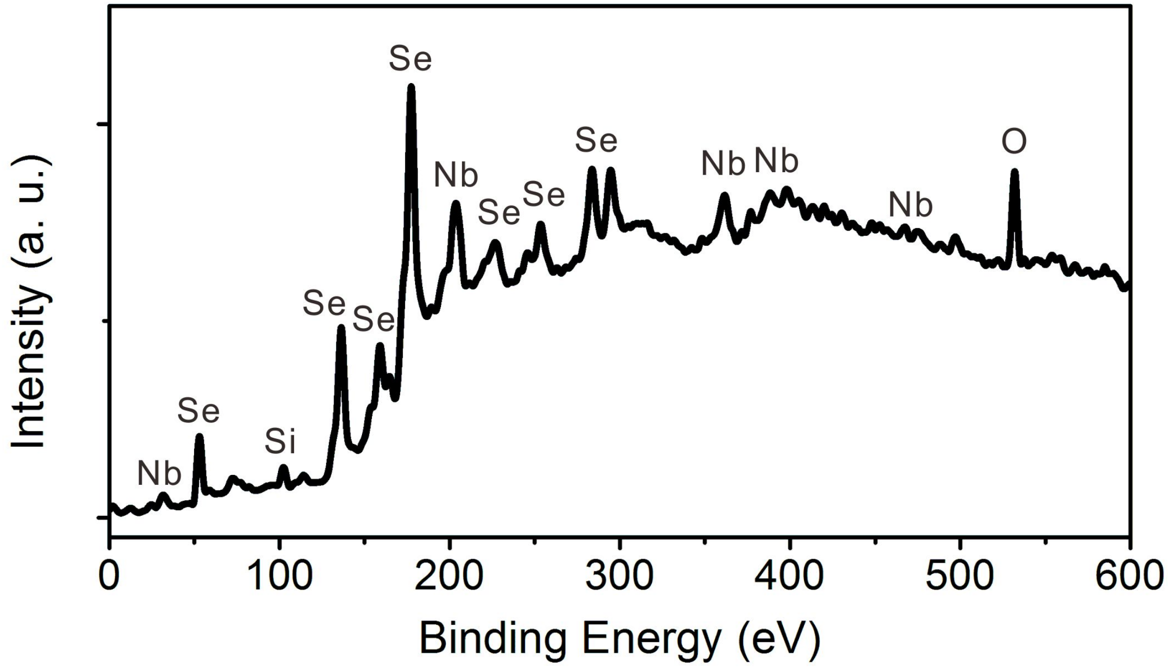

Supplementary Figure 5. XPS survey spectrum of few-layer $NbSe_2$ deposited on SiO_2/Si substrates

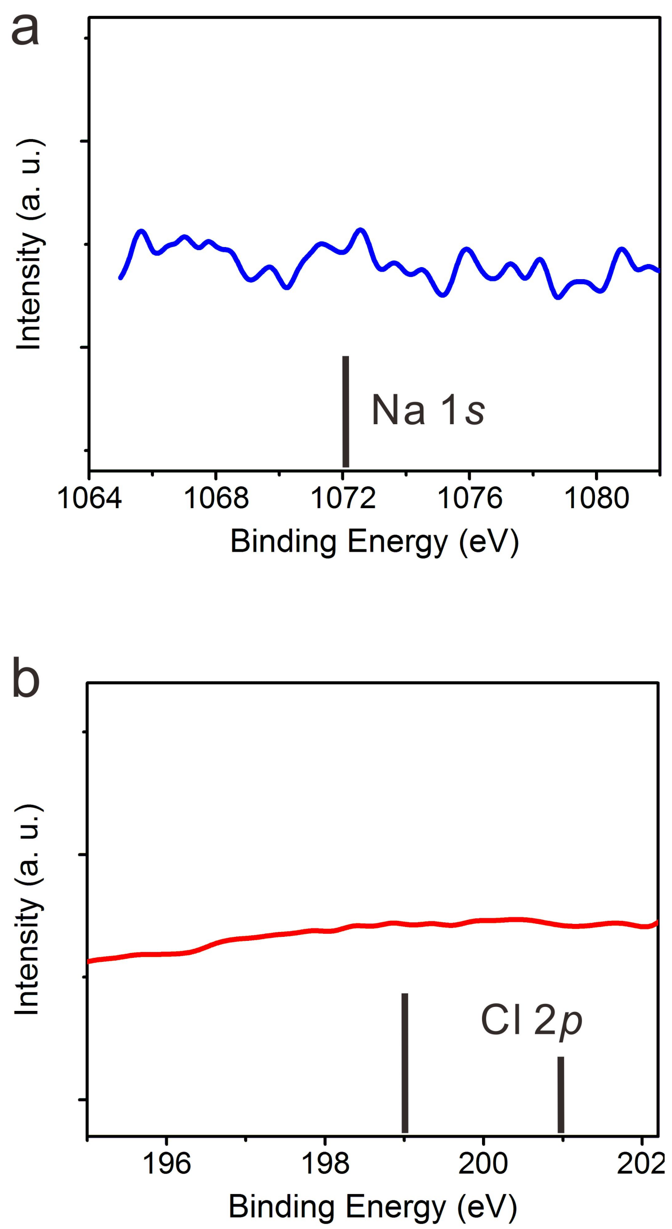

Supplementary Figure 6 XPS characterization of Na and Cl residues in the as-grown $NbSe_2$. (a) Na 1*s* and (b) Cl 2*p* XPS spectra collected on $NbSe_2$ samples. For comparison, standard Na 1*s* and Cl 2*p* line positions are shown in the figure. No Na 1*s* and Cl 2*p* peaks are observed in the XPS spectra, indicating as-grown $NbSe_2$ sample are free of Na and Cl contamination.

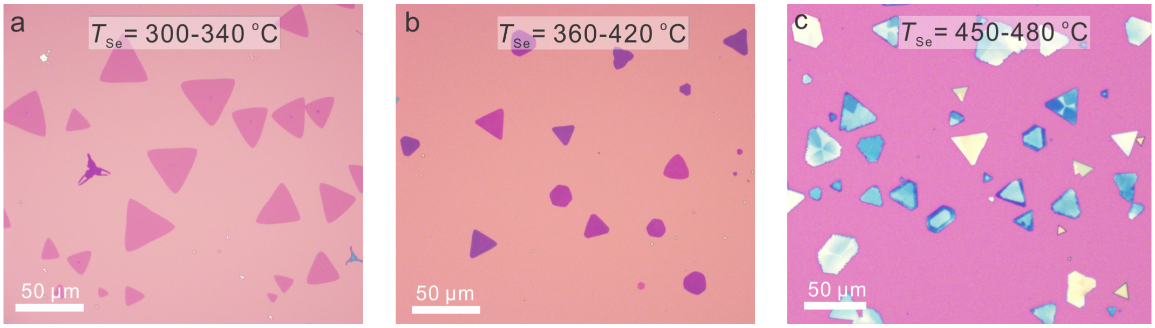

Supplementary Figure 7. Representative morphologies of $NbSe_2$ crystals synthesized with T_{Se} setting at (a) 300-340 ºC, (b) 360-420 ºC and (c) 450-480 ºC, respectively.

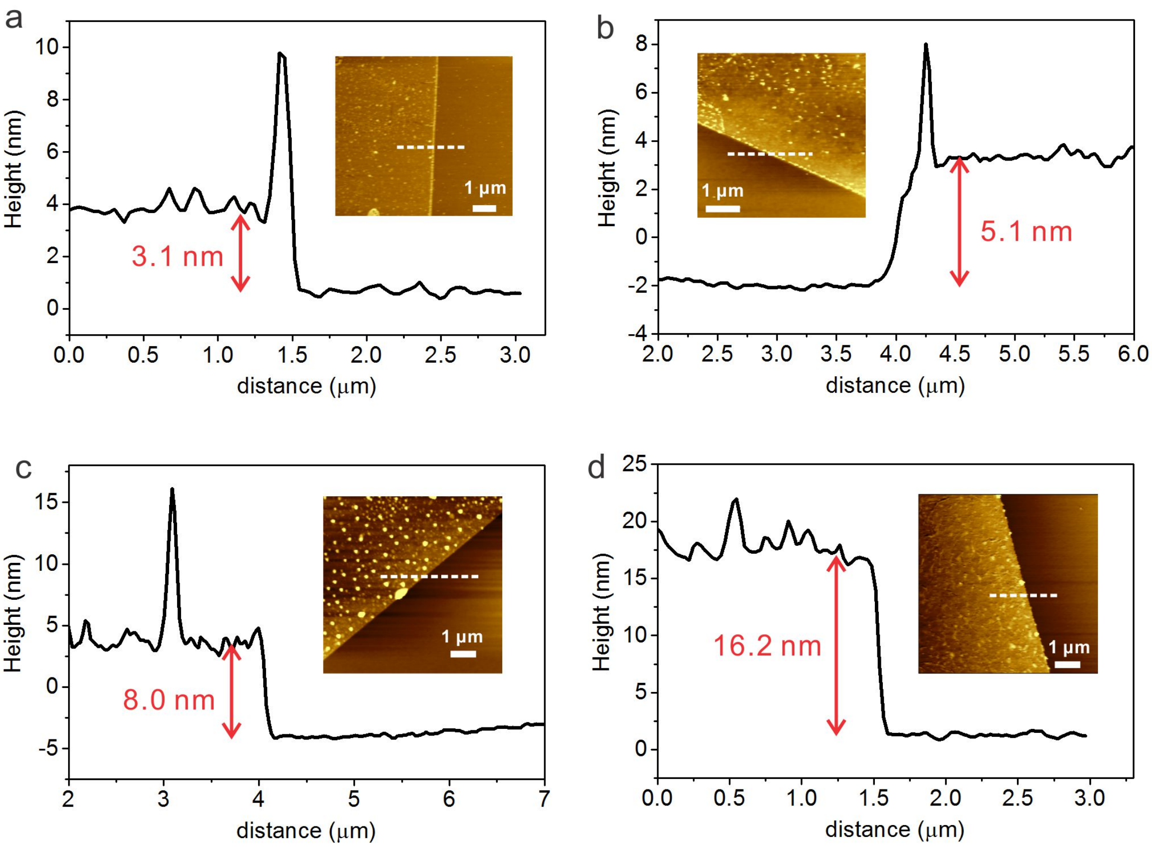

Supplementary Figure 8. AFM height profiles and images (inset) of $NbSe_2$ with different thickness.

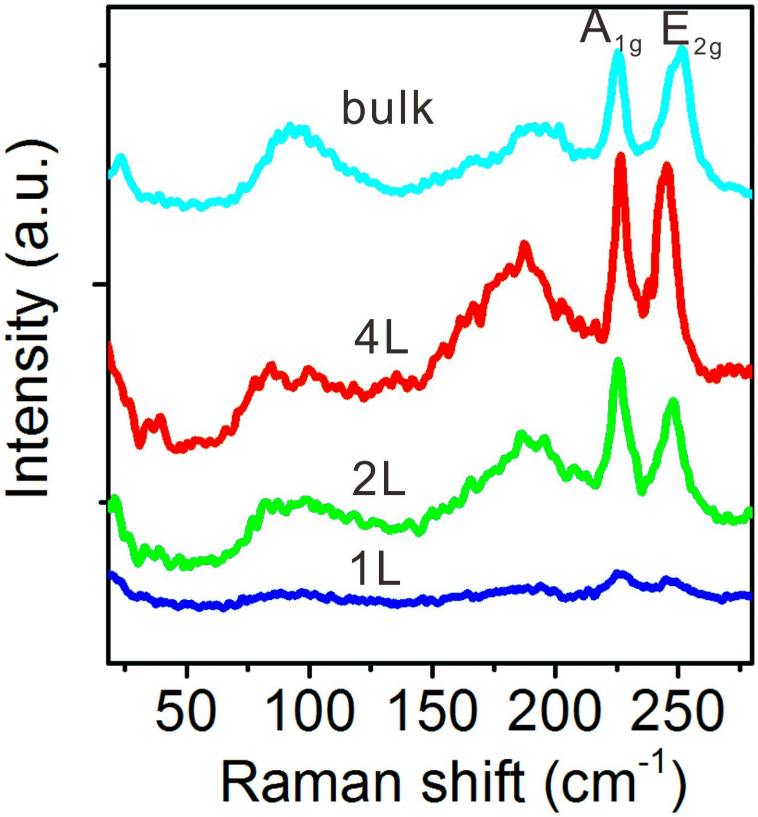

Supplementary Figure 9. Room-temperature Raman spectra of $NbSe_2$ with different thickness.

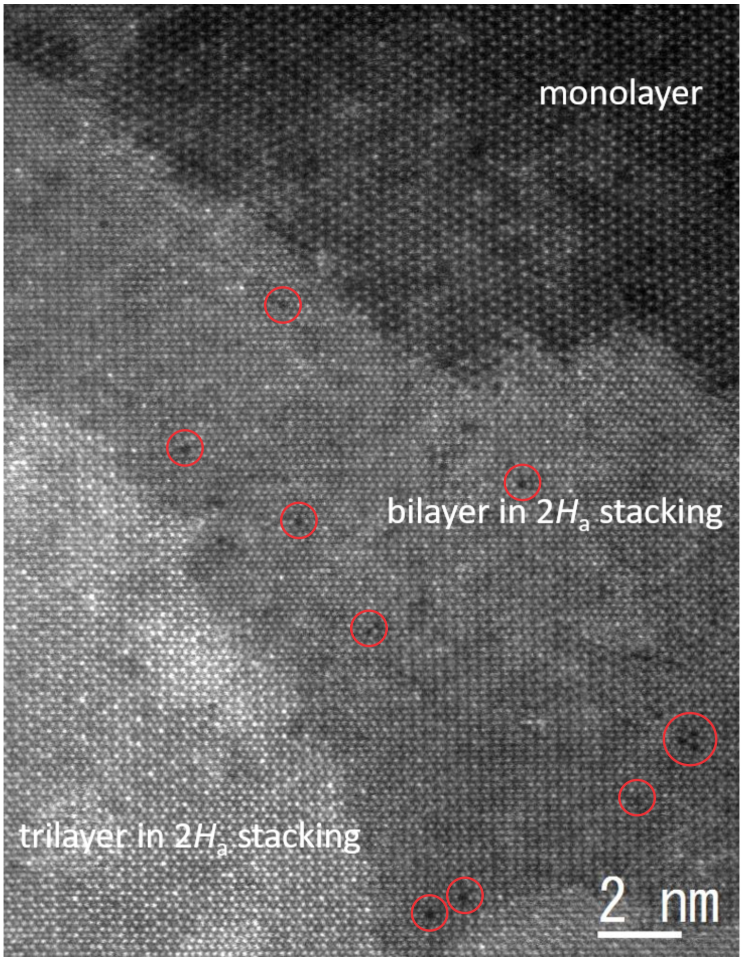

Supplementary Figure 10. ADF-STEM image of the multiple layer region of $NbSe_2$. The stacking of the CVD-grown sample is found to be mostly in $2H_a$ stacking, while a few regions show $2H_c$ stacking. The major defect types in multiple layer $NbSe_2$ are Se vacancies (indicated by red circles), similar to the case in monolayer $NbSe_2$.

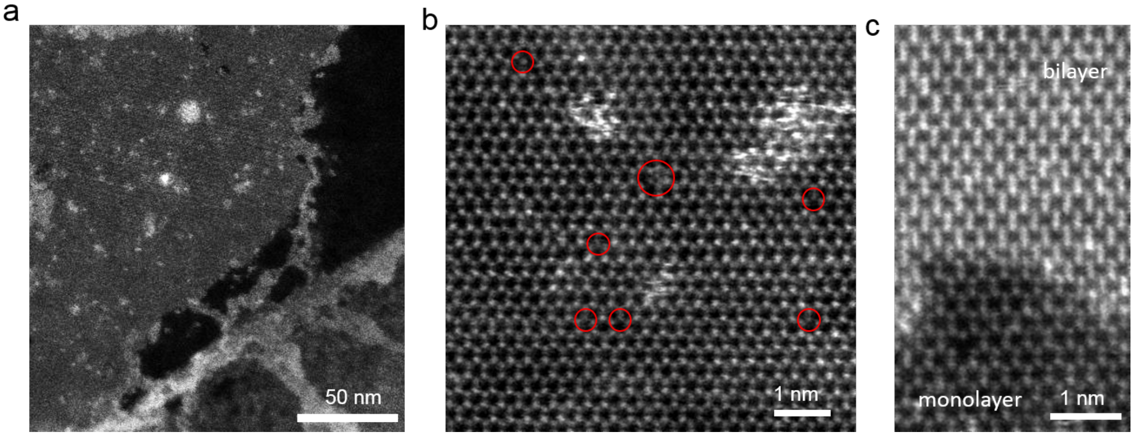

Supplementary Figure 11. ADF-STEM images of monolayer $NbSe_2$ grown directly on graphene. (a) Low magnified ADF-STEM image showing a large region of monolayer $NbSe_2$. (b) Atomic resolution ADF-STEM image of the high-quality hexagonal $NbSe_2$ lattice. Point defects are indicated by the red circles. Most of the defects are Se vacancy, similar to the case grown on SiO_2/Si substrate. (c) Atomic resolution ADF-STEM image showing a step edge of the bilayer $NbSe_2$ film with the $2H_c$ stacking.

Supplementary Note 1: Superconductivity of few-layer NbSe2 crystals

The superconductivity was also observed in few-layer $NbSe_2$ crystals. Supplementary Figure 12a shows the superconductivity observed on Sample B with the 10-layer $NbSe_2$. Similar to that observed in Sample A in the main text, the sample shows a metallic behavior at high temperatures. With the temperature further reduced, R_{xx} drops at onset temperature T_{onset} = 5.5K and reaches zero resistance at T_{zero} = 4.2K. Compared to the thinner sample (Sample A), T_{zero} increases from 0.8K to 4.2K. From the H_{c2}-T_c phase diagram shown in Supplementary Figure 12b, the Ginzburg-Landau coherence length ξ_{GL} is estimated to be about 18nm. Supplementary Figure 12c shows the differential resistance dV/dI in zero magnetic field as a function of the bias current at different temperatures. The peaks in the dV/dI curve indicate the transition from the superconducting to the normal state. The peaks shift to zero with increasing temperature, and finally disappear at 5.1K. Supplementary Figure 12d shows the temperature dependence of the critical current I_c derived from Supplementary Figure 12c.

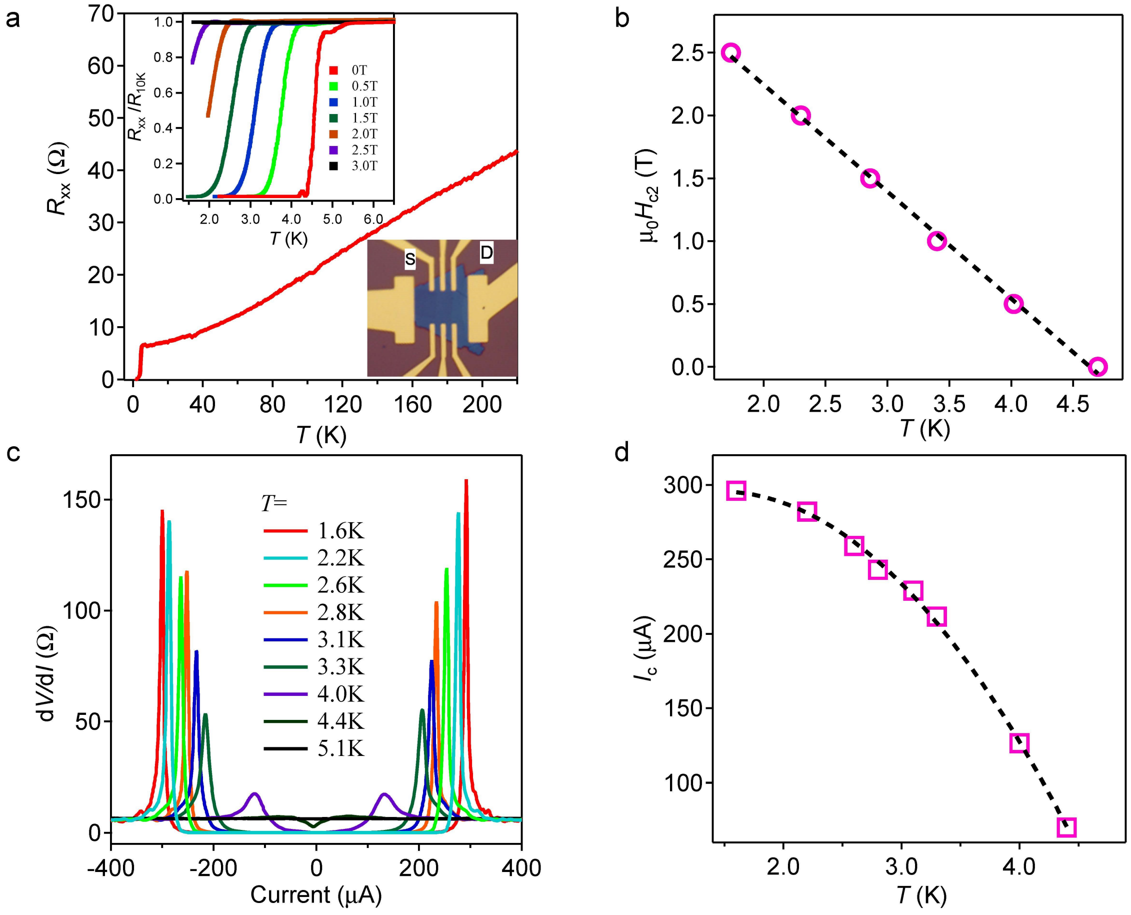

Supplementary Figure 12. Transport measurements on Sample B of a 10-layer $NbSe_2$ device. (a) Temperature dependence of the longitudinal resistance R_{xx} in zero magnetic field from 300K to 0.26K. Upper left inset: Superconductivity in different magnetic fields. Lower right inset: Optical image of a typical 10-layer $NbSe_2$ device. (b) Temperature dependence of the upper critical field H_{c2}. The dashed line is the linear fit to H_{c2}. (c) Differential resistance in zero magnetic field as a function of the bias current at different temperatures. (d) Temperature dependence of the critical current. The dashed line is a guide to the eye.

Supplementary Note 2: Influence of oxygen and moisture on the superconductivity of few-layer $NbSe_2$

The influence of oxygen and moisture on the CVD-grown $NbSe_2$ was carried out by exposing samples (#1 and #2) to ambient conditions with different time followed immediately by measuring their transport properties. Supplementary Figure 13 shows the typical experimental results collected on Sample #1. To characterize the changes of sample quality, the residual resistance ratio *RRR*, the critical temperature T_c ($0.5R_N$), and the superconducting transition width ΔT_c are summarized in Supplementary Table 1. As the exposure time increases from 0h to 24h, *RRR* decreases from 3.82 to 3.56 and T_c ($0.5R_N$) reduces from 4.94K to 4.21K. Correspondingly, the superconducting transition broadens gradually from 0.37K to 1.05K. For Sample #1, the superconductivity can still be observed when it was exposed to air for 24 hours. However, when the exposure time increases to 36 hours, no superconductivity can be detected and a metal-insulator transition emerges around 170K as shown in Supplementary Figure 13b. No conductive behavior was found when the sample was further exposed to air. From Supplementary Table 1, Sample #2 seems to be much more stable and the superconductivity can survive with 36 hours exposure. These data indicate that, even the $NbSe_2$ samples are capped with graphene, they are still sensitive to oxygen and moisture. The ambient degrades the samples with increasing exposure time.

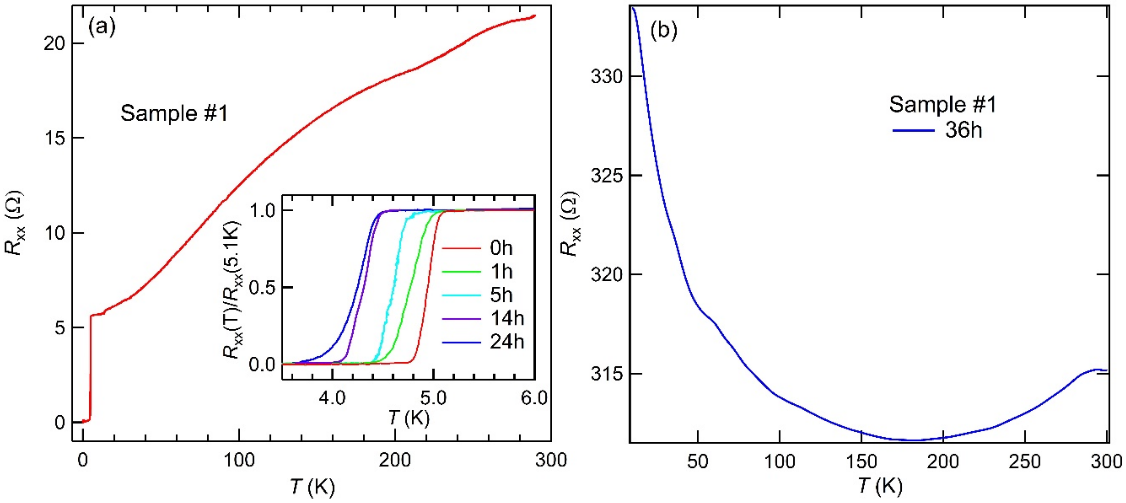

Supplementary Figure 13. Ambient-exposure effects of superconductivity in CVD-grown $NbSe_2$. (a) Temperature dependence of resistivity for $NbSe_2$ exposed with different time from 0h to 24h (indicated by different colors). (b) *R*-*T* plot for the same $NbSe_2$ sample after 36h exposure to ambient.

Supplementary Table 1. Residual resistance ratio *RRR*, critical temperature T_c (0.5R_N), and superconducting transition width ΔT_c for CVD-grown few-layer $NbSe_2$ samples exposed to ambient

Sample	Ambient exposure time	*RRR*	T_c (K)	ΔT_c (K)
Sample #1	0h	3.82	4.94	0.37
	1h	3.63	4.74	0.64
	5h	-	4.60	0.58
	14h	3.46	4.30	0.67
	24h	3.56	4.21	1.05
Sample #2	0h	3.15	3.25	0.70
	1h	3.14	3.25	0.65
	5h	3.15	3.24	0.50
	14h	2.95	3.0	0.42
	19h	2.81	2.82	1.42
	36h	2.57	2.73	1.73

Supplementary Note 3: Superconductivity of few-layer $NbSe_2$ grown on graphene substrate

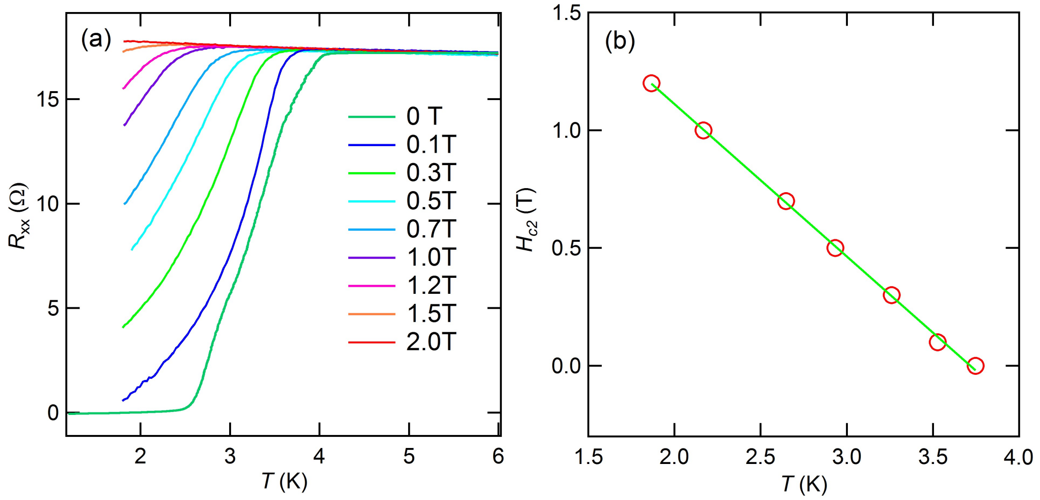

Supplementary Figure 14. Superconductivity in 8-layer thick $NbSe_2$ grown on Graphene substrate. (a) Temperature dependence of the resistance under different magnetic fields. (b) Temperature dependence of the upper critical field H_{c2}. The solid line is a linear fit to H_{c2}.

The superconductivity of few-layer $NbSe_2$ grown on graphene substrate was also investigated. The transport data of an 8-layer $NbSe_2$ sample grown on graphene is shown in Supplementary Figure 14. At zero magnetic field, the superconducting transition critical temperature T_c of an 8-layer $NbSe_2$ on graphene is ~3.2K. The superconducting transition ΔT_c is about 1.5K, which is wider than the few-layer (5-layer and 10-layer) $NbSe_2$ grown on SiO_2/Si substrate. These observations could be caused by the presence of PMMA residues on transferred CVD graphene film. The residues on graphene may act as nucleation centers for $NbSe_2$ growth. Therefore, more defects may exist in the $NbSe_2$ crystals grown on graphene substrates, resulting in poorer superconducting properties.

Supplementary Note 4: Estimation of carrier density and mean free path for monolayer superconducting $NbSe_2$

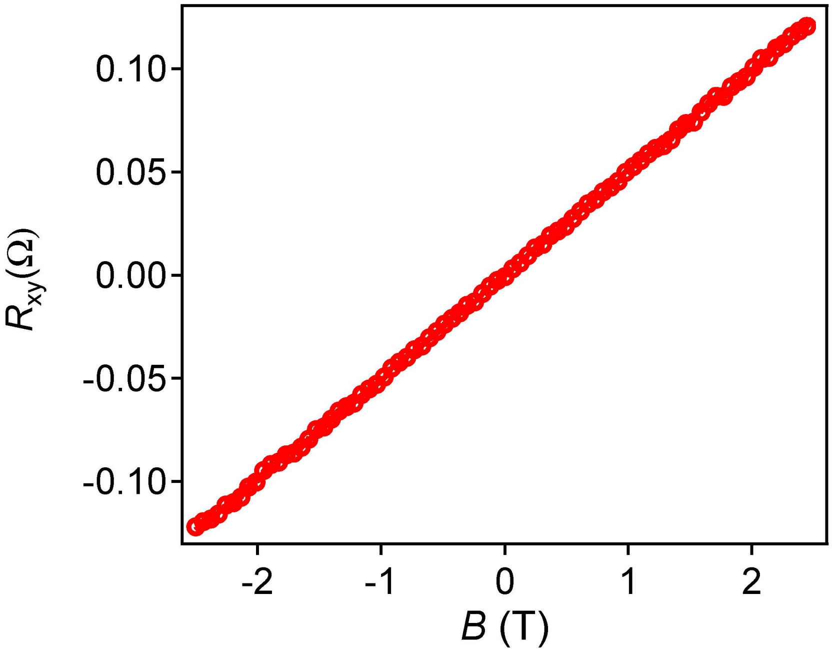

Supplementary Figure 15. Hall resistance R_{xy} measured at 2.0 K for a monolayer $NbSe_2$ crystal. Measurements were performed in the normal state with a current bias of 1 μA on the crystals while the magnetic field was applied perpendicularly to the sample plane. The carrier density and mean free path were estimated to be 1.25×10^{16} cm^{-2} and 1.3 nm by using the following procedure.

1) Estimation of carrier density n_s

By fitting the experimental data in Supplementary Figure 15, we can calculate the carrier density n_s via the following formula:

$$n_s = \frac{I/e}{\mathrm{d}V_H/\mathrm{d}B} = \frac{1/e}{\mathrm{d}R_H/\mathrm{d}B} = 1.25\times10^{16}\,\mathrm{cm}^{-2} \quad (1)$$

2) Estimation of sheet resistance R_s

The width w and length l for the measured sample can be determined by the optical image. The normal state resistance R_N = 242.3 Ω was taken at T = 1.5 K. Then the sheet resistance R_s can be calculated via the following formula:

$$R_s = \frac{R_N w}{l} = 727\ \Omega\ \square^{-1} \quad (2)$$

3) Estimation of mobility μ

According to Drude model, the mobility μ can be calculated via the following formula:

$$\mu = \frac{1}{eR_s n_s} = 0.69\ \mathrm{cm^2V^{-1}s^{-1}} \quad (3)$$

4) Estimation of the Fermi velocity v_F

In two-dimensional systems, the Fermi velocity v_F can be calculated via the following formula:

$$v_F = \frac{\hbar}{m}\sqrt{2\pi n_s} = 3.25\times10^6\ \mathrm{m\ s^{-1}} \quad (4)$$

5) Estimation of the momentum scattering time τ_m

$$\tau_m = \frac{\mu m}{e} = 3.9\times10^{-16}\,\mathrm{s} \quad (5)$$

6) Estimation of the mean free path l_m

$$l_m = v_F \tau_m = 1.3\ \mathrm{nm} \quad (6)$$

7) Estimation of the Fermi wave vector k_F

$$k_F = \sqrt{2\pi n_s} = 2.8\times10^8\ \mathrm{cm^{-1}} \quad (7)$$

8) Estimation of the Ioffe-Regel criterion

$$k_F l_m = 37 \quad (8)$$

Supplementary Note 5: Comparison of $NbSe_2$ and some high-T_c superconductors

It is instructive to consider together $NbSe_2$ and some high-T_c cuprates and Fe-based superconductors (FeSCs)[1-5]. Structurally, while they all crystallize in layered structures, they possess different superconductively active layers including $NbSe_2$ layer in $NbSe_2$, CuO_2 planes in cuprates and Fe_2As_2/Fe_2Se_2 layers in FeSCs. While un-doped $NbSe_2$ is a superconductor, the superconductivity in cuprates and FeSCs emerges upon doping carriers into antiferromagnetic parent compounds[2]. Despite their diversity in structure, the phase diagrams of these materials share the common characteristic that superconductivity exists near the boundary of an ordered phase (CDW in $NbSe_2$[6], spin/charge density wave in cuprates and iron arsenides[1, 3, 7]). The presence of various nearby magnetic and other competing orders in cuprates and FeSCs makes it difficult to understand the relationship between ordered phases and superconductivity[7, 8]. By comparison, $NbSe_2$ is a much simple model, in which the competitive CDW and superconductivity order is believed to be induced by pure electron–phonon coupling[8]. Therefore, the research on $NbSe_2$ may provide insight into the understanding of high-T_c superconductivity.

On the other hand, the 2D superconductivity in high-T_c superconductors is seldom studied due to the difficulty in ultrathin sample preparation. FeSe as the simplest FeSCs has been successfully synthesized by MBE. The most striking finding in monolayer FeSe is the significant enhancement of T_c when grown on a $SrTiO_3$ substrate[9, 10]. However, the superconductor-substrate interaction in $NbSe_2$ system has not been investigated. With our proposed CVD technology, we grew $NbSe_2$ layers on graphene substrates and the transport measurement indicates a decrease of T_c compared with SiO_2/Si substrate (see Supplementary Note 3). This phenomenon indicates that the substrate also plays important role in determining the superconductivity of ultrathin $NbSe_2$. Based on the developed CVD method, more investigations could be done to understand the 2D superconductor-substrate interplay, as well as to explore the unexpected and novel superconductivity of $NbSe_2$ on specific substrates.

Supplementary References

1. Orenstein, J. & Millis, A. Advances in the physics of high-temperature superconductivity. *Science* **288**, 468-474 (2000).
2. Kamihara, Y., Watanabe, T., Hirano, M. & Hosono, H. Iron-Based Layered

Superconductor La[O1-xFx]FeAs (x = 0.05−0.12) with Tc = 26 K. *Journal of the American Chemical Society* **130**, 3296-3297 (2008).

3. Canfield, P.C. & Bud'Ko, S.L. FeAs-based superconductivity: a case study of the effects of transition metal doping on BaFe2As2. *Annu. Rev. Condens. Matter Phys.* **1**, 27-50 (2010).
4. Miyata, Y., Nakayama, K., Sugawara, K., Sato, T. & Takahashi, T. High-temperature superconductivity in potassium-coated multilayer FeSe thin films. *Nat Mater* **14**, 775-779 (2015).
5. He, Q.L. et al. Two-dimensional superconductivity at the interface of a Bi2Te3/FeTe heterostructure. *Nature Communications* **5**, 4247 (2014).
6. Xi, X. et al. Strongly enhanced charge-density-wave order in monolayer NbSe2. *Nat Nano* **10**, 765-769 (2015).
7. Tranquada, J., Sternlieb, B., Axe, J., Nakamura, Y. & Uchida, S. Evidence for stripe correlations of spins and holes in copper oxide superconductors. *Nature* **375**, 561 (1995).
8. Kiss, T. et al. Charge-order-maximized momentum-dependent superconductivity. *Nat Phys* **3**, 720-725 (2007).
9. Ge, J.-F. et al. Superconductivity above 100 K in single-layer FeSe films on doped SrTiO3. *Nat Mater* **14**, 285-289 (2015).
10. Tan, S. et al. Interface-induced superconductivity and strain-dependent spin density waves in FeSe/SrTiO3 thin films. *Nature materials* **12**, 634-640 (2013).
